# Continuing genomic evolution of the *Neisseria meningitidis* cc11.2 urethritis clade, *Nm*UC: a narrative review

**DOI:** 10.1099/mgen.0.001113

**Published:** 2023-10-18

**Authors:** Emilio I. Rodriguez, Yih-Ling Tzeng, David S. Stephens

**Affiliations:** ^1^​ Division of Infectious Diseases, Department of Medicine, Emory University School of Medicine, Atlanta, GA, USA

**Keywords:** antimicrobial resistance, genomic evolution, *Neisseria gonorrhoeae*, *Neisseria meningitidis*, *Nm*UC, urogenital pathogen

## Abstract

*Neisseria meningitidis (Nm*) is a bacterial pathogen responsible for invasive meningococcal disease. Though typically colonizing the nasopharynx, multiple outbreaks of meningococcal urethritis were first reported in 2015–2016; outbreaks originally presumed to be caused by *Neisseria gonorrhoeae* (*Ng*). Genomic analysis revealed that the *Nm* isolates causing these outbreaks were a distinct clade, and had integrated gonococcal DNA at multiple genomic sites, including the gonococcal denitrification apparatus *aniA–norB*, a partial gonococcal operon of five genes containing *isp*D, and the acetylglutamate kinase gene *argB* with the adjacent gonococcal locus *NGO0843*. The urethritis isolates had also deleted the group C capsule biosynthesis genes *cssA/B/C* and *csc*, resulting in loss of capsule. Collectively, these isolates form the *N. meningitidis* urethritis clade (*Nm*UC). Genomic analysis of recent (2016–2022) *Nm*UC isolates revealed that the genomic features have been maintained in the clade, implying that they are important for *Nm*UC’s status as a urogenital pathogen. Furthermore, the analysis revealed the emergence of a sub-clade, designated *Nm*UC-B, phylogenetically separated from the earlier *Nm*UC-A. This sub-clade has integrated additional gonococcal alleles into the genome, including alleles associated with antimicrobial resistance. *Nm*UC continues to adapt to a urethral niche and evolve as a urogenital pathogen.

## Data Summary

All isolate genomes/sequences used in this paper are available on the PubMLST database (https://pubmlst.org/organisms/neisseria-spp). The PubMLST isolate data are provided in Table S1 (available in the online version of this article).

**Impact Statement**

This study analyses the genomic evolution of the *Neisseria meningitidis* (*Nm*) cc11.2 urethritis clade, *Nm*UC. *Nm*UC has caused multiple outbreaks of meningococcal urethritis reported from 2015 to 2022; outbreaks that were originally presumed to be caused by *Neisseria gonorrhoeae *. Previous genomic analysis revealed that *Nm*UC isolates have integrated gonococcal DNA at multiple genomic sites. This review establishes that these gonococcal genomic features of the clade have been maintained in the majority of the *Nm*UC isolates, suggesting their importance in the clade. This review further reveals the emergence of an *Nm*UC sub-clade that has acquired additional alleles, including those conferring antimicrobial resistance. *Nm*UC is continuing to evolve as a pathogen.

## Introduction

*Neisseria meningitidis* (*Nm*) and *Neisseria gonorrhoeae* (*Ng*), both exclusively human pathogens, are closely related species that share a common ancestor but have evolved to occupy distinct human ecological niches. *Nm,* a colonizer of the human nasopharynx, is the cause of invasive meningococcal disease (IMD), usually presenting as meningitis, bacteraemia, or sepsis [1, 2]. Prior to the widespread introduction of new meningococcal conjugate and protein-based vaccines, an estimated 1.2 million cases of meningococcal disease and a death toll of approximately 135 000 occurred annually [3]. The case fatality rate is 10–15 %, and 11–19 % of survivors experience long-term consequences such as neurological disabilities, sensory impairment/loss, and limb or digit loss [4]. IMD has been declining worldwide due in part to the introduction of these new effective vaccines. At the beginning of the coronavirus disease 2019 (COVID-19) pandemic in 2020, stringent infection control measures resulted in a further drop in IMD cases, with country case reductions ranging from 27–91 % of pre-pandemic levels [5]. However, in 2022–2023, as COVID-19 control measures waned, IMD has increased [6–10].

The asymptomatic nasopharyngeal *Nm* carriage rate in different populations varies from 5–40 % in an age-dependent manner [11]. Approximately 10 % of adults are asymptomatic nasopharyngeal carriers of *Nm* in non-epidemic periods [12, 13] and this percentage increases in certain populations: 30 % in men who have sex with men (MSM) and 24 % in adolescents [14–18]. *Nm* can also colonize the rectum [14, 19]. Antibiotic resistance is emerging in *Nm* to penicillin (including beta-lactamase-producing strains) and fluoroquinolones such as ciprofloxacin [20–22].

*Ng*, a major sexually transmitted disease pathogen causing over 80 million cases globally, infects the mucous membranes of the reproductive tract, cervix, uterus, and fallopian tubes in women, and the urethra in women and men. *Ng* can also infect the mucous membranes of the mouth, throat, eyes, and rectum [19, 23, 24]. Antibiotic resistance in *Ng* has become a major threat to treatment regimens [25].

Genetic analyses suggest that *Ng* may have evolved from a meningococcal clone that acquired the capacity to colonize the genital tract [26, 27]. In the last two decades, outbreaks of IMD, believed to be sexually transmitted, have occurred among MSM in North America and also in Europe, usually caused by clonal complex 11 (cc11) isolates expressing group C capsule [6, 7, 28–33]. Sporadic cases of meningococcal urethritis have also been previously reported [34], some associated with the MSM cc11 group C outbreaks. However, beginning in 2015 multiple distinct outbreaks of *Nm* male urethritis cases, originally attributed to *Ng*, were recognized primarily in heterosexual men [35]. The colonization of the nasopharynx by *Nm* suggests oral sex may be the transmission route for urethral meningococcal infection [36]. Phylogenetic analysis of cc11 isolates showed that the sexually transmitted *Nm* urethritis isolates, designated as the *Nm* urethritis clade, *Nm*UC [35, 37], form a distinct branch within the lineage 11.2 of cc11 and the closest relatives were among the cc11 isolates from invasive cases [37, 38].

The Centers for Disease Control and Prevention (CDC) has confirmed over 400 cases of meningococcal urethritis in multiple states of the USA. A total of 209 USA urethritis isolates (collected 2013–2016) belonging to the closely related *Nm*UC (primarily isolated from heterosexual males) were sequenced and characterized [35, 37]. Subsequently, additional *Nm*UC isolates were recovered from cases in the USA, the UK, Japan, and Vietnam; many of the Vietnamese isolates were collected from MSM urethritis patients. This emerging *Nm*UC appears to be an effective male urethral pathogen, but has also been isolated from the oropharynx, rectum, and female genital tract, and been reported to cause neonatal conjunctivitis and, rarely, invasive disease [37, 39, 40]. Four of seven patients identified with IMD caused by *Nm*UC isolates had immunocompromising conditions, including HIV and complement deficiency [40]. The extent of the spread of the *Nm*UC has been significantly underestimated [41].

*Nm* and *Ng* are naturally competent bacteria and the genomes are known to be particularly plastic through frequent horizontal gene transfer; transformation is greatly enhanced by a specific DNA uptake sequence, widely distributed in both the *Nm* and *Ng* genomes [42]. To understand the emergence of *Nm*UC as a urogenital pathogen, the genomic signatures of the initial *Nm*UC isolates were determined [37, 38]. Analysis (summarized below) showed that a common ancestor of *Nm*UC underwent various homologous recombination events and acquired multiple fragments of gonococcal DNA [37]. Since this original analysis, 52 additional clade isolates (2016–2022) from diverse geographical sites have been added to the PubMLST database [43]. We performed phylogenomic analyses on all 261 clade isolates and showed a continued evolution of *Nm*UC, as well as the emergence of a sub-clade, designated *Nm*UC-B, a separate branch from the original *Nm*UC-A population. This review summarizes the overall genomic characteristics of the *Nm*UC that have persisted and the data supporting the potential biological roles of these genetic changes and describes additional genetic features that have arisen in the nearly decade-long proliferation and global spread of the *Nm*UC.

## Methods

### Isolates and genomes

All genomes in this study are available on the PubMLST Neisseria database [43]. PubMLST IDs and other relevant data are listed in Table S1. The *Nm*UC isolates used in this study were identified by the PubMLST Similar Isolates Identifier Tool. Briefly, isolates were selected with a mismatch threshold of 2 to the prototype clade isolate CNM3 (PubMLST #50559). Additional isolates were identified by the unique *Nm*UC alleles (*aniA*, *norB*, *ispD, argB*, *fHbp*) and IS1301-mediated *cps* deletion. Duplicate isolate records were removed.

### Phylogenomic analyses

Microreact (version 240) was used for phylogenomic analysis of the clade isolates [44]. On the Microreact plugin in PubMLST, all the 261 *Nm*UC isolates, along with 39 non-clade cc11 *Nm* isolates included as an outgroup, were analysed against the *N. meningitidis* cgMLST v2 scheme. The phylogenetic tree was rooted with the 39 non-clade isolates.

### Genomic analyses

The PubMLST Genome Comparator tool was used to determine the specific gene alleles of isolates [43]. Harvest suite (v1.1.2) was used to identify novel genomic features in *Nm*UC-B [45]. Clade whole-genome sequences (both *Nm*UC-A and -B) were downloaded from the PubMLST database. Genomes were aligned using the core genome multi-aligner Parsnp (v1.2) and then visualized with Gingr (v1.2). Aligning the *Nm*UC-A to *Nm*UC-B genomes revealed SNP regions that were characteristic of the sub-clade. Regions of SNPs were identified by examining the genome alignments, and the affected loci were noted. The affected loci were analysed in all clade isolates with the Genome Comparator (set to rescan undesignated loci), and their gene alleles and allele frequency were noted. The percentage identity of loci was determined by comparing the nucleotide sequences through blast (v2.13.0) against the NCBI’s non-redundant nucleotide collection database using megablast [46]. The species and percentage identity of the best match were provided.

## Genomic features of *Nm*UC

**Fig. 1.**
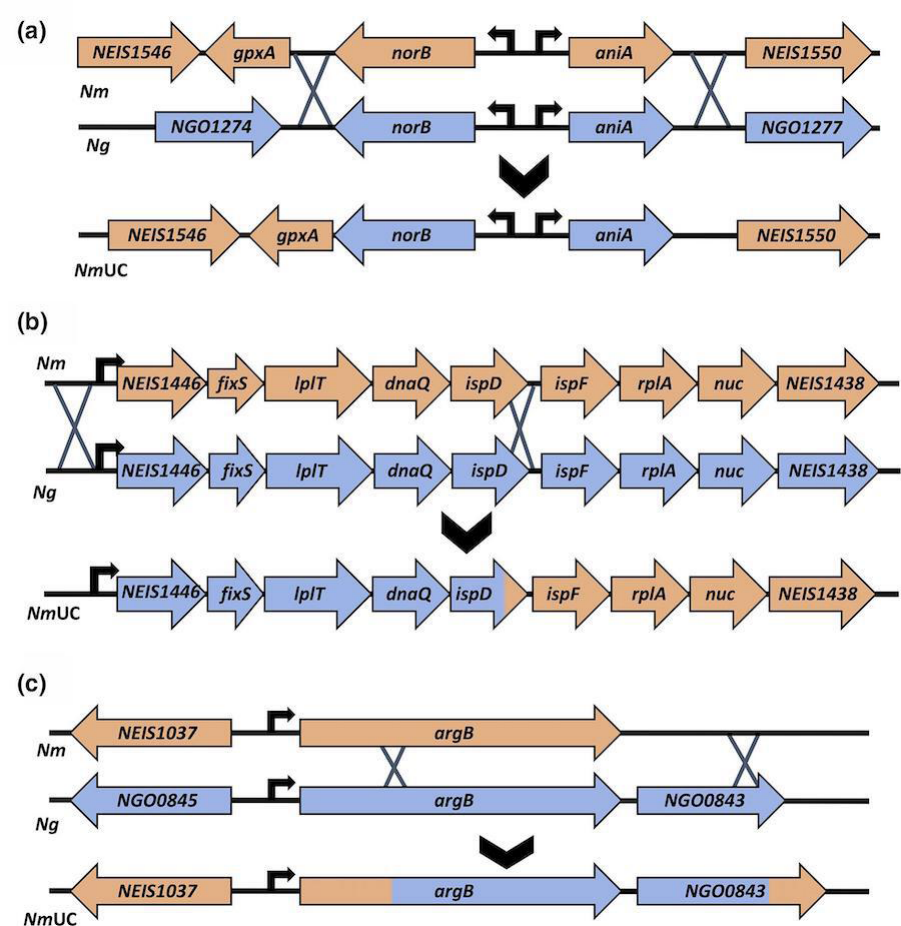
(**a**) The gonococcal denitrification apparatus in *Nm*UC. The *Nm* ancestor of *Nm*UC underwent homologous recombination with *Ng* DNA, integrating the gonococcal *norB-aniA* cassette into the genome, bolstering denitrification and microaerobic respiration. Panel (a) is a modification of Fig. 2a in [38]. (**b**) The gonococcal *isp*D operon in *Nm*UC. The homologous recombination with *Ng* DNA integrated five genes (*ispD* was partial recombined) of a larger nine-gene operon into the genome and acquired gonococcal alleles. (**c**) The *argB* and *NGO0843* genes in *Nm*UC. The gonococcal *argB* and *NGO0843* (encoding a hypothetical protein) genes were integrated into the *Nm* ancestor genome. The two loci were only partially recombined, and the integration of *NGO0843* resulted in an alternative stop codon and a longer coding sequence than that found in *Ng*.

**Fig. 2.**
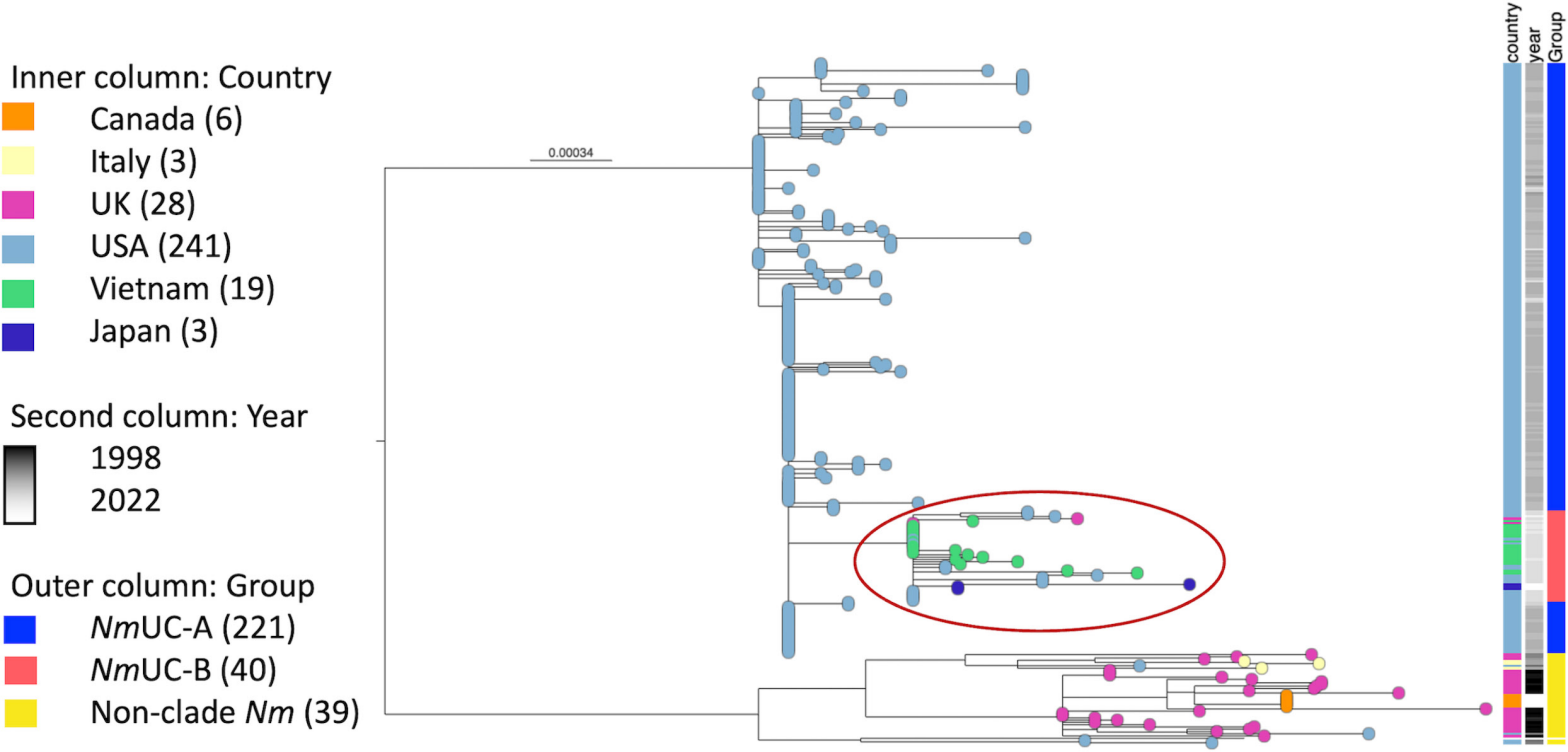
SNP-based phylogeny of *Nm*UC isolates. *Nm*UC isolates (*n*=261) and a comparison group of non-clade lineage 11.2 *Nm* (*n*=39) are included. Isolates are marked at the tip and inner column for the country of origin, the second column for year of isolation and the outer column for the grouping of isolates. The *Nm*UC-B branch in the phylogenetic tree is also outlined in red to highlight this emerging sub-clade. In the legend, the bracketed numbers denote the number of isolates in each category.

**Fig. 3.**
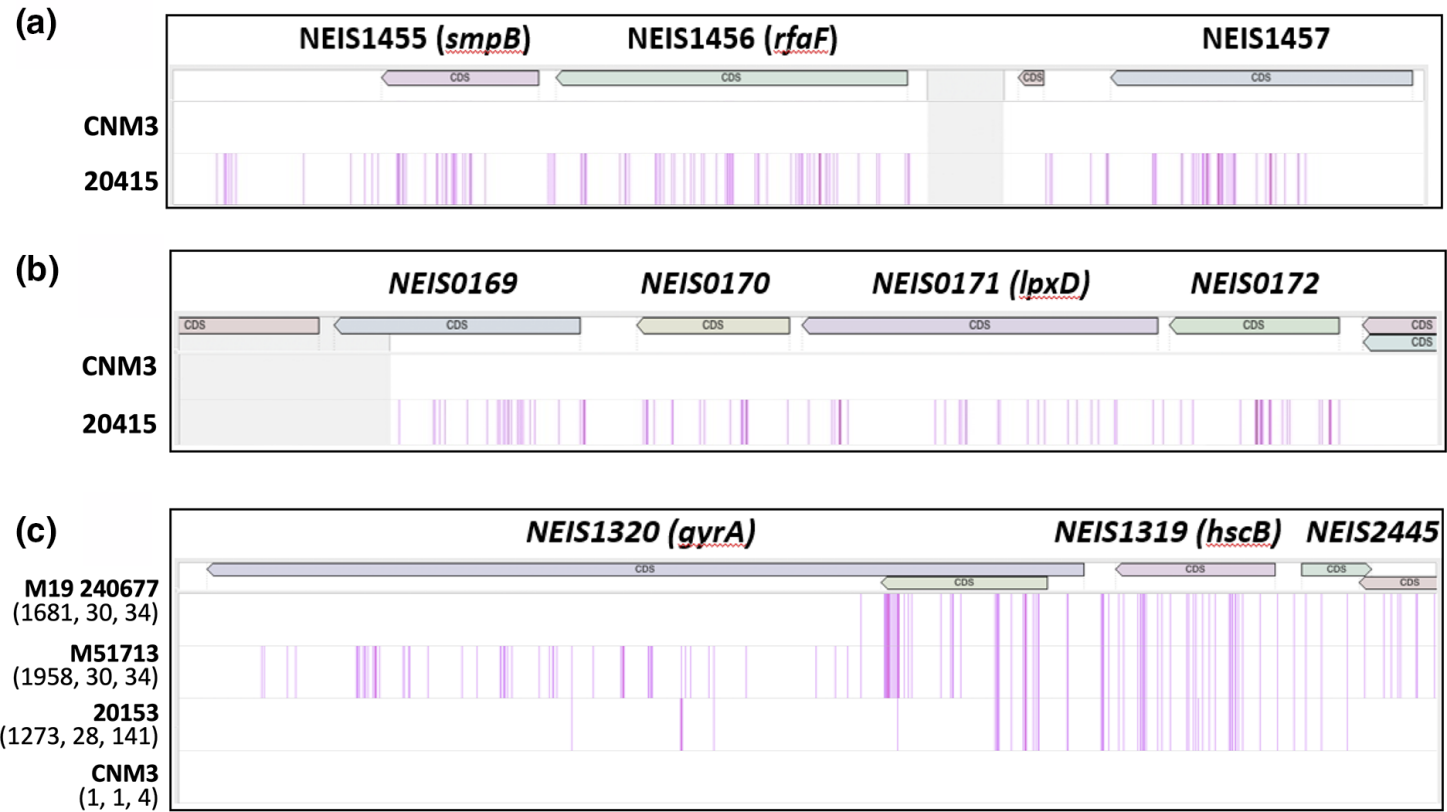
SNP density plots of new SNP regions defining the 40 *Nm*UC-B sub-clade isolates. Core genomes were aligned with isolate CNM3 set as the reference genome, as CNM3 has the standard (most abundant) alleles for each of the new characteristic regions of *Nm*UC-B. Each SNP that differs from CNM3 is shown as a single line, and multiple neighbouring SNPs appear as thick lines. The light-grey region indicates that sequence is absent in one or more of the aligned genomes. One representative region from each category in Table 3 is shown. (**a**) Region A, present in all 40 *Nm*UC-B isolates. (**b**) Region G, present in most*Nm*UC-B isolates. (**c**) Region L, present in a significant minority. Three isolates that have varied allele combinations of NEIS1320, NEIS1319 and NEIS2445 are plotted with allele numbers included, respectively, in parentheses.

Two hundred and nine *Nm*UC isolates were included in the original clade genomic analysis by Retchless *et al*. [37]. Of these isolates, 195 were collected from the male urogenital system, 3 from the female urogenital system, 5 from sterile sites (blood or cerebrospinal fluid), 3 from the nasopharynx, 2 from the eyes, and 1 from the rectum. SNP-based phylogeny revealed the closest relatives of *Nm*UC were invasive capsular group C cc11 isolates. Time-measured Bayesian phylogeny modelling found that *Nm*UC was estimated to have diverged from the closely related invasive disease isolates in 2006, and the most recent common ancestor (MRCA) of the clade existed in 2011 [37]. The *Nm*UC MRCA contained 7.6 kb of *Ng* DNA, integrated in three distinct genomic regions: (1) the denitrification cassette *norB-aniA* (Fig. 1a), (2) a partial operon of five genes, including *lplT*, *dnaQ,* and *ispD* (Fig. 1b), and (3) *argB* encoding acetylglutamate kinase along with the adjacent gonococcal hypothetical protein *NGO0843* (Fig. 1c) [37, 38]. Furthermore, additional recombination events occurred with DNA of *Ng* and commensal *Neisseria *, resulting in varied amounts of transferred DNA in individual *Nm*UC isolates ranging from 5.7 to 30.2 kb [37]. Whole-genome sequencing (WGS) also revealed that all clade isolates have replaced group C capsule genes, *cssA/B/C* and *csc*, with an insertion element *IS1301*, and consequently inactivated capsule expression [37, 38]. Furthermore, *Nm*UC was found to express a unique factor H-binding protein (FHbp) variant; FHbp is a virulence protein that enhances evasion of the human host immune system [47]. These initial key genomic features of the clade are expanded upon below.

### Loss of capsule

Capsules are a virulence factor relevant for evading the human immune system; most *Nm* invasive isolates from blood or cerebrospinal fluid express a polysaccharide capsule outside the cell envelope [48]. Based on the antigenic structures of capsular polysaccharides and genetic configurations of the capsular polysaccharide locus (*cps*), meningococci are divided into 12 serogroups. *Nm*UC has an insertion of *IS1301* into the *cps* locus. Specifically, the *IS1301* insertion deleted the sialic acid biosynthesis genes *cssA*/*B*/*C* (NEIS0054–NEIS0052) and 620 bp of the capsule polymerase gene *csc* (NEIS0051), making the clade non-encapsulated and thus nongroupable [37, 38]. This is a universal feature of all *Nm*UC clade isolates. While capsule is important for IMD, meningococci carried at mucosal surfaces often express reduced capsule or are non-encapsulated; capsules impede meningococcal attachment to mucosal surfaces [49]. Gonococci also do not produce capsule, suggesting that the loss of capsule in *Nm*UC may enhances attachment to urogenital epithelial cells [49] and may benefit urogenital colonization [27, 37, 38].

### Microaerobic/anaerobic growth

When colonizing the nasopharynx, *Nm* aerobically respires with oxygen as an electron acceptor. In contrast, the human urethra is a microaerobic/anaerobic environment where colonizing bacteria experience oxygen limitation [50, 51]. Gonococci colonize the urethra and survive using denitrifying respiration through nitrite reductase AniA (NEIS1549), which catalyses the conversion of nitrite to nitric oxide (NO), and nitric oxide reductase NorB (NEIS1548), which subsequently reduces NO to nitrous oxide [52]. These two proteins enable the utilization of nitrite and NO as alternative respiratory electron acceptors [52], and gonococci universally have functional AniA and NorB. In contrast, many *Nm* strains have mutated *aniA* and/or *norB*, or completely lack the *aniA* gene [52–54] and thus are unable to support anaerobic growth [53, 54]. The recombination event of a~3.7 kb fragment of gonococcal DNA replaced the *Nm norB–aniA* locus (Fig. 1a), resulting in an *aniA* (allele 204) and a *norB* (allele 753), which are unique to *Nm*UC and have 1171/1173 nucleotides identical to the gonococcal *aniA* allele 42 (e.g. in FA1090) and 2255/2256 nucleotides identical to the gonococcal *norB* allele 356. The genetic conversion of the denitrification pathway, which also brought in the gonococcal intergenic region (IGR) controlling the divergently transcribed *aniA* and *norB*, is not present in non-clade *Nm* isolates [55–57]. The *Ng norB*/*aniA* allele pair 753/42 was found in 197/209 of the *Nm*UC clade isolates, and 5 more isolates have alleles with only a single SNP in either *aniA* (1 isolate) or *norB* (4 isolates). A recent study showed that oxygen consumption, nitrite utilization, and NO production were significantly altered by the *Ng* AniA–NorB conversion in the *Nm*UC, resulting in different denitrifying aerobic and microaerobic growth of the *Nm*UC. Denitrification and microaerobic respiration were bolstered and protection against host-derived NO likely enhanced, supporting the *Nm*UC adaptation and survival in a microaerobic urogenital environment [38, 58]. These genes have been implicated in biofilm formation during natural gonococcal infection [59], and NorB also plays a significant role in protection against NO, produced by epithelial and phagocytic host cells encountered during infection. Hence, the AniA–NorB denitrification pathway plays a crucial role in gonococcal growth and pathogenesis during urogenital infection. Overall, recombination of the gonococcal denitrification genes appears to contribute to *Nm*UC’s ability to colonize the urethra [37, 38].

### Other alleles

The second recombination of gonococcal DNA into the clade genome involved a partial operonNEIS1446–NEIS1442, a 3.3 kb *Ng* segment altering five genes of a larger nine-gene operon (Fig. 1b). The recombination event changes the entire coding sequence of our affected genes, resulting in 100 % identity to *Ng* orthologues: a conserved hypothetical protein (NEIS1446), the *cbb3*-type cytochrome oxidase maturation protein *fixS* (NEIS1445), the lysophospholipid transporter gene *lplT* (NEIS1444) and the DNA polymerase III subunit epsilon gene *dnaQ* (NEIS1443). The fifth gene, *ispD* (NEIS1442), encoding the 2-C-methyl-d-erythritol 4-phosphate cytidyltransferase that is part of the terpenoid mevalonate-independent (MEP) pathway, had 588/691 nucleotides (85 %) replaced from the 5′ end by the recombination event, resulting in 98.5 % identity to *Ng ispD* [37, 38]. While the same NEIS1446 and *fixS* alleles are present in all 209 isolates, *lplT* (allele 44), *dnaQ* (allele 328), and *ispD* (allele 302) are not conserved in all clade isolates (Table 1). Two isolates appeared to have undergone another recombination event altering their *lplT/dnaQ/ispD* (alleles 842, 500, and 567, respectively), and these distinct alleles are not found in any other *Neisseria* genomes in PubMLST. The consequence of this gene conversion event in the clade remains to be defined, but preliminary characterization [60] suggested that the gonococcal IspD may affect microaerobic/anaerobic growth of *Nm*UC.

**Table 1.** Frequencies of alleles in the initial 209 (2013–2016)^¢^ *Nm*UC-A isolates

Gene	Allele no.	No. of isolates	No. of SNPs¶
*NEIS1549 (aniA)*	204	201	0
	375	7	87
	205	1	1
*NEIS1548 (norB)*	753	198	0
	1236	7	57
	1237	3	1
	1239	1	1
*NEIS1446 (HP)^∗^*	27	209	0
*NEIS1445 (fixS)*	95	209	0
*NEIS1444 (lplT)*	44	202	0
	840	1	1
	842^∗∗^	2	13
	Undefined†	4	1
*NEIS1443 (dnaQ)*	328	199	0
	329	6	1
	499	2	1
	500^∗∗^	2	29
*NEIS1442 (ispD)*	302	207	0
	567^∗∗^	2	52
*NEIS1038 (argB)*	351	209	0
*NGO0843*	Undefined‡	205	0
*fHbp*	1127	205	0
	Undefined§	2	1
	1146	1	1
	1237	1	14

The PubMLST Genome Comparator tool was used to determine the specific gene alleles of isolates [43].

^∗^HP, hypothetical protein.

†Undefined *lplT* alleles in all four isolates have the same frameshift mutation.

‡*NGO0843* does not have a defined NEIS number/allele. A total of 205 isolates have identical *NGO0843* sequences, while the remaining 4 isolates have various SNP differences.

§Undefined *fHbp* alleles have internal stop codons.

¶The numbers of SNPs are determined relative to the most abundant alleles.

^∗∗^Alleles marked with a double asterisk are present in the same two isolates.

¢Denotes the years the isolates were collected.

The third conserved gonococcal recombination event involves *argB* (NEIS1038), encoding the acetylglutamate kinase, a key enzyme for arginine biosynthesis. This event resulted in 583 bp of *Ng* DNA integrating into the 3′ end of *argB* locus (897 bp) (Fig. 1c), resulting in a unique *argB* (allele 351) present in all 209 *Nm*UC isolates [37]; allele 351 is only present in *Nm*UC. This recombination event also partially involves the adjacent hypothetical protein *NGO0843*, a locus not present in *Nm* and with no defined NEIS number (Fig. 1c). The integration of only the 5′ end of *NGO0843* resulted in a stop codon further downstream and a longer coding sequence than in *Ng* (288 vs 357 bp). This specific *NGO0843* allele is present in 205/209 of the original *Nm*UC isolates.

Lastly, the clade has acquired a unique FHbp variant, likely via a recombination event with other meningococcal DNA [37]. The majority (205/209) of original clade isolates have *fHbp* allele 1127, which is unique to *Nm*UC (Table 1) [37, 38]. FHbp is a key antigen in meningococcal serogroup B-directed vaccines, which are being investigated as a potential method of protection against *Ng*.

## Persistence of genomic changes in recent *Nm*UC isolates (2017–2021)

Since the original studies defining the *Nm*UC, the clade has continued to be identified and expand geographically. Along with the original 209 isolates, as of September 2023 an additional 52 clade isolates have been recorded in the PubMLST database [43]. These new isolates have been collected from 6 states in the USA (28 isolates) as well as the UK (2), Vietnam (19), and Japan (3) (Table S1) [61–63]. As detailed below, analysis of the 52 recent (2016–2022) clade isolates indicates the emergence of a sub-clade *Nm*UC-B, but persistence of the original genomic signatures of the clade is observed.

Key genomic features remain present in the *Nm*UC. First, the deletion of capsule genes *cssA/B/C* and partial deletion of *csc* by *IS1301*, as well as the gonococcal *argB* allele (allele 351), were found in all 261 clade isolates. Second, the gonococcal *norB-aniA* cassette also remains a characteristic of the clade, though with additional changes. Of the 52 recent isolates, 47 retain both the gonococcal *aniA* and gonococcal *norB* as originally defined (alleles 204/753) (Table 2). One isolate has a single synonymous SNP in *aniA*, and two isolates have no identified *norB* in their genome sequence, likely due to poor WGS quality. The remaining two isolates (invasive 2019 USA isolates) contain an *Nm aniA* allele 675, commonly found in group E *Nm*, and an *Nm* IGR, but *Ng norB* allele carrying two silent SNPs relative to allele 753. The presence of the gonococcal NEIS1446–NEIS1442 genes also continues to be a characteristic of the clade; 51/52 of the recent isolates retain the operon as originally described (Table 2), with the *Ng* homologues of NEIS1446, *fixS*, *dnaQ,* and *ispD* remaining unaltered in all of the new clade isolates. One isolate has an *lplT* allele 1877 that differs from the standard clade allele 44 by a single SNP, resulting in a serine-to-leucine mutation. Lastly, the characteristic *NGO0843* clade allele is present in 46/52 of the recent isolates, and *fHbp* allele 1127 is present in all recent isolates. The persistence of the *Ng* genes in the clade suggests that these genes remain relevant to the clade’s evolution as a urogenital pathogen.

**Table 2.** Allele frequency of characteristic *Nm*UC genes in the 52 recent (2016–2022)^¢^ clade isolates

Gene	Allele no.	No. of isolates	No. of SNPs‡
*NEIS1549 (aniA)*	204	49	0
	675	2	85
	873	1	1
*NEIS1548 (norB)*	753	48	0
	2246	2	2
	Undefined	2	§
*NEIS1446 (HP)†*	27	52	0
*NEIS1445 (fixS)*	95	52	0
*NEIS1444 (lplT)*	44	51	0
	1877	1	1
*NEIS1443 (dnaQ)*	328	52	0
*NEIS1442 (ispD)*	302	52	0
*NEIS1038 (argB)*	351	52	0
*NGO0843*	Undefined^∗^	46	0
*fHbp*	1127	52	0

The PubMLST Genome Comparator tool was used to determine the specific gene alleles of isolates [43].

^∗^ *NGO0843* does not have a defined NEIS number/allele. A total of 46 isolates have identical *NGO0843* sequences, while the remaining 6 isolates have various SNP differences.

†HP, hypothetical protein.

‡The numbers of SNPs are determined relative to the most abundant allele.

§The two isolates without defined PubMLST allele numbers have, respectively, one SNP (as compared to allele 753) and no identified *norB* sequence.

¢Denotes the years the isolates were collected.

## Emerging *Nm*UC-B sub-clade

SNP-based phylogeny was performed on the 261 *Nm*UC isolates to determine additional genomic differences that have arisen based on time and/or geographical location. Furthermore, 39 non-clade cc11 *Nm* isolates were added to provide a genomic outgroup in the analysis because they are in the same clonal complex as *Nm*UC, including 26 non-cladeurogenital isolates and 13 invasive group C isolates (Table S1). The resulting phylogenetic tree revealed the emergence of a sub-clade within *Nm*UC (Fig. 2). Since 2019, *Nm*UC have been identified in countries outside the USA, including 2 isolates from the UK, 3 from Japan, and 19 from a Vietnam outbreak. These isolates clustered together with 16/20 more recent *Nm*UC isolates from the USA collected from 2019 to 2022, forming a sub-clade of 40 isolates, designated as *Nm*UC-B (Fig. 2). Importantly, all 19 Vietnamese isolates and both UK isolates were collected from MSM, as opposed to heterosexual men, as was initially typical of *Nm*UC-A [61, 62]. In addition to the original genomic features (deletion of capsule genes, acquisition of *Ng* denitrification apparatus, acquisition of the partial *Ng NEIS1446–NEIS1442* operon and *argB/NGO0843*), genomic alignment revealed new genetic characteristics of the sub-clade that are absent in *Nm*UC-A.

Several new genetic features, defined by blocks of continuous SNPs, were present in all of the 40 sub-clade isolates (Table 3). These include a 2.9 kb fragment encoding NEIS1455–NEIS1457 (Fig. 3a) and a 1.3 kb fragment that includes NEIS1590–NEIS1592 and NEIS3123. Both fragments have 100 % identity to *Ng* sequences. Of the loci covered by the 2.9 kb fragment, NEIS1455 and NEIS1456 have 100 % identity to *Ng* DNA and NEIS1457 has 97.5 % homology to *Ng*. For the 1.3 kb fragment, NEIS1591 and NEIS1592 have 100 % identity to *Ng*, whereas NEIS1590 and NEIS3123 were only partially replaced. In addition, a 2.0 kb region covering NEIS1609 (*folP*)–NEIS1610, a 1.2 kb region covering NEIS1611 and NEIS1613, and a 3.1 kb region covering NEIS1807–NEIS1809 have clusters of SNPs with the highest homology to non-gonococcal *Neisseria* DNA, including sequences from non-clade *Nm* and *Neisseria cinerea. N. cinerea* is a commensal that may be found in the urogenital tract of humans [64]. These unique gene blocks are all universally present in the sub-clade. In contrast, *Nm*UC-A has typical *Nm* sequences for genes mentioned above.

**Table 3.** Identity and frequency of characteristic new SNP regions in the 40 *Nm*UC-B sub-clade isolates

Region	Length (kb)	Region identity§	Gene affected	Allele no.	No. of SNPs^∗^	Gene identity§	No. of Isolates	Putative function
**Present in all**								
A	2.9	*Ng* (100 %)	*NEIS1455 (smpB)*	3	24	*Ng* (100 %)	40	SsrA-binding protein
			*NEIS1456 (rfaF)*	43	54	*Ng* (100 %)	40	Heptosyltransferase II
			*NEIS1457*	765	40	*Ng* (97.5 %)	40	Putative methylated-DNA–protein-cysteine methyltransferase
B	1.3	*Ng* (100 %)	*NEIS1590*	509	1	*Nm* (99.8 %)	40	Putative lipoprotein
			*NEIS1591*	3	30	*Ng* (100 %)	40	DNA-3-methyladenine glycosylase I
			*NEIS1592*	9	17	*Ng* (100 %)	40	Putative lipase
			*NEIS3123*	129	1	*Nm* (100 %)	40	Citrate (Si) synthase
C	2.0	*Nc*† (96.6 %)	*NEIS1609 (folP)*	1330	61	*Nm* (97.2 %)	40	Dihydropteroate synthase
			*NEIS2494*	Undefined¶	n/a	n/a	40	Putative phospho-2-dehydro-3-deoxyheptonate aldolase
			*NEIS1610*	2156	202	*Nm* (98.8 %)	40	Hypothetical protein
D	1.2	*Nm* (95.2 %)	*NEIS1611*	Undefined¶	n/a	n/a	40	3-octaprenyl-4-hydroxybenzoate carboxy-lyase
			*NEIS1613*	483	25	*Nm* (96.6 %)	40	Hypothetical protein
E	3.1	*Nm* (95.95 %)	*NEIS1807*	276	10	*Nl^‡^* (97.5 %)	40	Hypothetical protein
			*NEIS1808 (ampG)*	1057	56	*Nm* (96.8 %)	40	Putative integral membrane signal transducer protein
			*NEIS1809 (glnA)*	979	69	*Nm* (96.2 %)	40	Glutamine synthetase
**Present in majority**								
F	2.7	*Ng* (100 %)	*NEIS0103*	942	20	*Ng* (98.4 %)	38	Methionyl-tRNA formyltransferase
			*NEIS0104*	25	50	*Ng* (100 %)	36	SUN family protein
			*NEIS0105*	6	22	*Ng* (100 %)	36	Hypothetical protein
			*NEIS0106*	3277	49	*Nm* (97.7 %)	33	Putative two-component sensor kinase
G	2.8	*Ng* (99.6 %)	*NEIS0169*	159	23	*Ng* (99.6 %)	37	Hypothetical protein
			*NEIS0170*	17	16	*Ng* (100 %)	37	(3R)-hydroxymyristoyl-ACP dehydratase
			*NEIS0171 (lpxD)*	974	25	*Ng* (99.7 %)	34	UDP-3-O-[3-hydroxymyristoyl] glucosamine N-acyltransferase
			*NEIS0172*	393	21	*Ng* (99.6 %)	34	Putative outer membrane protein
H	2.0	*Nm* (99.7 %)	*NEIS1552*	36	13	*Nm* (100 %)	38	Hypothetical protein
			*NEIS1553 (lptA)*	1216	10	*Nm* (99.4 %)	37	Lipid A phosphoethanolamine transferase
I	1.1	*Nc*† (98.3 %)	*NEIS1307 (clpX)*	794	78	*Nc^†^* (97.3 %)	37	ATP-dependent protease ATP-binding subunit
**Present in Significant minority**								
J	1.9	*Ng* (100 %)	*NEIS1385*	1401	40	*Nm* (98.4 %)	12	ATP-dependent DNA helicase
			*NEIS1386*	13	41	*Ng* (100 %)	12	DNA polymerase IV
K	2.2	*Ng* (99.95 %)	*NEIS2110 (sstT)*	8	16	*Ng* (100 %)	4	Serine/threonine transporter
				10	15	*Ng* (100 %)	3	
				1647	11	*Ng* (99.7 %)	1	
			*NEIS3168*	68	7	*Ng* (100 %)	8	Hypothetical protein
			*NEIS2112*	14	38	*Ng* (100 %)	4	Putative outer membrane protein
				2660	16	*Ng* (98.9 %)	3	
				2665	27	*Ng* (99.5 %)	1	
L	2.4	*Ng* (98.3 %)	*NEIS1320 (gyrA)*	1273	20	*Nm* (99.6 %)	8	DNA gyrase subunit A
				1958	92	*Ng* (99.9 %)	2	
				1681	38	*Ng* (98.7 %)	1	
			*NEIS1319 (hscB)*	28	29	*Ng* (100 %)	8	Chaperone protein
				30	28	*Ng* (100 %)	3	
			*NEIS2445*	141	3	*Ng* (99.6 %)	8	Hypothetical protein
				34	4	*Ng* (100 %)	3	

Affected loci alleles and their frequency in clade isolates were determined with the PubMLST Genome Comparator [43]. The percentage identity of loci was determined by blast [46].

^∗^The numbers of SNPs are determined relative to the ‘standard‘ clade allele, the most abundant allele in *Nm*UC-A.

†*Nc, Neisseria cinerea.*

‡*Nl, Neisseria lactamica.*

§Gene may only be partially covered by SNP region, and SNP region identity may not dictate gene identity.

¶Undefined alleles are the result of truncated coding sequence or gene deletion.

Several additional features were present in the majority, but not all, of *Nm*UC-B sub-clade isolates. A 2.7 kb region covering NEIS0103–NEIS0106 with 100 % homology to *Ng* is present in 30/40 isolates that contain a unique allele combination for these genes (Table 3). NEIS0103 has 98.4 % identity to *Ng* DNA, and NEIS0104 and NEIS0105 have 100 % identity to *Ng* DNA. The remaining 10 isolates carry different alleles in 1 or 2 genes. A 2.8 kb region covering 4 genes, NEIS0169–NEIS0172, with 99.64 % homology to the corresponding *Ng* sequence, is consistent in 34 isolates and all 4 loci have over 99.6 % identity to *Ng* DNA (Fig. 3b). Two additional non-*Ng* genetic features are present in the majority of sub-clade isolates. A 2.0 kb region of SNPs covering NEIS1552–NEIS1553 (*lptA*) is present in all but three isolates and has over 99 % identity to non-clade *Nm*. A 1.1 kb region containing a single gene, NEIS1307 (*clpX*), shows 98.3 % homology to that of *N. cinerea* and is present in all but three isolates.

Lastly, several polymorphisms are only present within a minority (≤12) of sub-clade isolates, suggesting possible local clonal expansion (Table 3). Nine isolates collected in New City York in 2019 as well as three isolates collected in Japan in 2022 have NEIS1385 allele 1401 and NEIS1386 allele 13; while in the rest of the sub-clade isolates and the majority of *Nm*UC-A (239 isolates), both loci are allele 1. A 2.2 kb region of SNPs covering NEIS2110, NEIS3168, and NEIS2112 has 99.9 % homology to *Ng*, and is present in eight isolates, four of which were collected in New York City in 2019 while the other four were collected in Vietnam in 2019–2020. Subsequent SNP changes have occurred in this region, resulting in altered alleles, but the presence of the 2.2 kb *Ng* sequence remains consistent in these eight isolates. For example, four isolates have NEIS2110 allele 8, three have allele 10 (which differs from allele 8 by one SNP), and one has allele 1647 (which differs from allele 8 by five SNPs). For NEIS2112, 4 isolates have allele 14, 3 have allele 2660 (which differs from allele 14 by 24 SNPs), and 1 has allele 2665 (which differs from allele 14 by 11 SNPs). In the rest of the sub-clade and the majority of *Nm*UC-A, the NEIS2110 allele is 475. Finally, a 2.4 kb region of SNPs covering NEIS1320 (*gyrA*), NEIS1319, and NEIS2445 (Fig. 3c) with 98.3 % homology to *Ng* is present in eight isolates collected from Vietnam in 2019–2020. There are three more isolates, two collected in the USA and one from the UK in 2019, containing alleles of NEIS1319 and NEIS2445, with only one nucleotide difference from those of the eight Vietnam isolates. These 3 regions cover 8 genes and 16 different alleles, of which 14 have the highest homology to *Ng* DNA.

## Antibiotic resistance in *Nm*UC

In contrast to *Ng*, widespread multidrug resistance in *Nm* has remained rare, although resistance is increasing. *Nm* is naturally highly resistant to the model antimicrobial peptide polymyxin B (PmB, MIC 64–256 µg ml^−1^); the *Nm*UC isolates demonstrated stable subpopulations of heteroresistant colonies that showed near total resistance to PmB (MIC 384–1024 µg ml^−1^) and colistin (MIC 256 µg ml^−1^), as well as enhanced LL-37 resistance [65]. Antimicrobial peptide (AMP) resistance in *Nm* was mainly due to active Mtr efflux and LptA-mediated lipid A modification. WGS, variant analyses and directed mutagenesis revealed that the heteroresistance phenotypes in *Nm*UC were the result of point mutations and IS*1655* element movement in the *pilMNOPQ* operon that encodes the type IV pilin biogenesis apparatus as it is hypothesized that inactivation of the pilin biogenesis apparatus further reduced entry of AMPs into the cell [65]. Cross-resistance to other classes of antibiotics was also observed in the heteroresistant derivatives [65].

*Nm* continues to be widely susceptible to a variety of antimicrobials [20]. The *Nm*UC clade remains susceptible to ceftriaxone and cefixime but has acquired alleles associated with decreased antibiotic susceptibility to penicillin, azithromycin, and ciprofloxacin, as detailed below [37, 62, 66]. Bazan *et al.* examined the antibiotic susceptibility of 122 *Nm*UC isolates collected from 2015 to 2019 in Columbus, Ohio, USA; all of the isolates had either intermediate penicillin susceptibility or were resistant (0.064–0.5 mg l^−1^) by E-test [66]. Chromosomally mediated penicillin resistance in *Ng* is attributed to five mutated resistance determinants (*penA*, *ponA*, *porB*, *mtrR* and *pilQ*); of these genes, the examined *Nm*UC isolates only display *penA* and *mtrR* alleles associated with resistance. In the overall clade collection, 259/261 isolates have *penA* allele 316, 1 isolate has the *penA* allele 327 and the remaining isolate has an undefined *penA* allele (Table 4). The *penA* allele 316 contains the following mutations linked to increased penicillin resistance: F504L, A510V, I515V, H541N, and I566V [66]; *penA* allele 327 has all those mutations as well, excluding I566V. Of the other resistance genes, a single *Nm*UC-A isolate collected in 2015 has an *mtrR* with an A86T mutation (allele 39) that is associated with increased azithromycin resistance in *Ng* [37, 67].

**Table 4.** Identity and frequency of alleles conferring increased antibiotic resistance in the 261*Nm*UC Isolates

Gene	Antibiotic	Allele no.	Gene identity	No. of isolates
*penA*	Penicillin	316	*Nm* (99.8 %)	259*
		327	*Ng* (100 %)	1
*‘mtrR*	Macrolide	39	*Ng* (98.6 %)	1
*gyrA*	Ciprofloxacin	9	*Ng* (100 %)	1
		140	*Ng* (100 %)	2
		381	*Nm* (98.1 %)	8
		382	*Nm* (98.1 %)	1
*NEIS1609 (folP)*	Sulfonamide	95	*Nm* (100 %)	3
		1330	*Nm* (97.2 %)	40

The PubMLST Genome Comparator tool was used to determine the specific gene alleles of isolates [43].

*The remaining one isolate has an undefined *penA* allele.

Additionally, evidence of emerging ciprofloxacin resistance is found in the sub-clade. Brooks *et al.* reported a ciprofloxacin-resistant *Nm*UC-B rectal isolate (MIC=0.38 µg ml^−1^) in the UK that had acquired a partial gonococcal *gyrA* allele 9 with T91F and D95A mutations that confer ciprofloxacin resistance (Table 4) [61]. Furthermore, eight of the *Nm*UC-B isolates from Vietnam contain the *gyrA* allele 381 (T91F and D95A) and an additional Vietnam isolate contains the *gyrA* allele 382 (T91I); these isolates have ciprofloxacin MICs between 0.19 and 3 µg ml^−1^ [62]. Finally, two sub-clade isolates collected from the USA in 2019 contain *gyrA* allele 140 (T91F and D95G), which is also associated with reduced ciprofloxacin susceptibility [37]. Thus, while none of the *Nm*UC-A isolates demonstrate reduced susceptibility to ciprofloxacin, 12/40 isolates in the more recently emerged *Nm*UC-B contain *gyrA* alleles associated with ciprofloxacin resistance. Evidence of emerging sulfonamide resistance is also found in in *Nm*UC-B. All 40 sub-clade isolates contain the *folP* allele 1330 (Table 4); this allele has the F31L, G194C, and R228S mutations, all associated with elevated sulfonamide MICs [68–71]. An additional three *Nm*UC-A isolates contain a *folP* allele 95 with the R228S mutation and a 195 S-196G insertion, also associated with sulfonamide resistance [68]. The *penA*, *gyrA,* and *folP* alleles in *Nm*UC suggest that the clade is acquiring antimicrobial resistance determinants and continuing to evolve as a pathogen.

## Conclusions

Historically, *Ng* likely evolved from a *Nm* clone acquiring the ability to colonize the urogenital tract [26, 27]. The recent evolution of *Nm*UC, resulting in tropism for the male urethra, is another example of the continuing evolution and adaption of *Nm*. The cc11.2 *Nm*UC clade was initially distinguished by the deletion of group C capsule genes and uptake of the gonococcal homologues of the AniA/NorB denitrification apparatus. These remain genomic signatures of the clade and likely contribute to the clade’s emergence as a urogenital pathogen. The contributions to urogenital pathogenicity of two other genomic signatures, the uptake of the gonococcal *NEIS1446–NEIS1442* containing *ispD* and the *Ng* acetylglutamate kinase gene *argB/NGO0843*, are less clear. However, these features have also been consistently maintained in the clade for nearly a decade, suggesting that they are also important for the adaptation and survival of the *Nm*UC in an uncommon human niche for *Nm*. Furthermore, the recent emergence of a sub-clade within *Nm*UC shows the clade continues to evolve. The sub-clade has acquired new genomic features, many of which are additional gene conversion events of homologues of gonococcal DNA. There is evidence of emerging antibiotic resistance in *Nm*UC-B to ciprofloxacin and sulfonamides. Resistance to antimicrobial peptides is also a key feature of the urogenital pathogenesis of *Ng* [25]. Thus, *Nm*UC isolates continue to undergo homologous recombination events with gonococcal DNA and acquire additional alleles that may contribute to *Nm*UC’s continued evolution as a urogenital pathogen.

## Supplementary Data

Supplementary material 1
